# Is suvorexant a better choice than alternative hypnotics?

**DOI:** 10.12688/f1000research.6845.1

**Published:** 2015-08-03

**Authors:** Daniel F. Kripke

**Affiliations:** 1 Scripps Clinic Viterbi Family Sleep Center, La Jolla, CA, USA

**Keywords:** suvorexant, Belsomra®, zolpidem, eszopiclone, melatonin, sleep, hypnotic, mortality, cancer

## Abstract

Suvorexant is a novel dual orexin receptor antagonist (DORA) newly introduced in the U.S. as a hypnotic, but no claim of superiority over other hypnotics has been offered.  The manufacturer argued that the 5 and 10 mg starting doses recommended by the FDA might be ineffective.  The manufacturer's main Phase III trials had not even included the 10 mg dosage, and the 5 mg dosage had not been tested at all in registered clinical trials at the time of approval.  Popular alternative hypnotics may be similarly ineffective, since the FDA has also reduced the recommended doses for zolpidem and eszopiclone.  The "not to exceed" suvorexant dosage of 20 mg does slightly increase sleep.  Because of slow absorption, suvorexant has little effect on latency to sleep onset but some small effect in suppressing wakening after sleep onset and in improving sleep efficiency. The FDA would not approve the manufacturer's preferred 40 mg suvorexant dosage, because of concern with daytime somnolence, driving impairment, and possible narcolepsy-like symptoms.  In its immediate benefits-to-risks ratio, suvorexant is unlikely to prove superior to currently available hypnotics—possibly worse—so there is little reason to prefer over the alternatives this likely more expensive hypnotic less-tested in practice.  Associations are being increasingly documented relating hypnotic usage with incident cancer, with dementia risks, and with premature death.  There is some basis to speculate that suvorexant might be safer than alternative hypnotics in terms of cancer, dementia, infections, and mortality.  These safety considerations will remain unproven speculations unless adequate long-term trials can be done that demonstrate suvorexant advantages.

## A new kind of hypnotic drug

The manufacturer has begun U.S. marketing for suvorexant (Belsomra®), a dual orexin receptor antagonist (DORA) offered as a new hypnotic for treatment of insomnia (See Table 1 for abbreviations). The manufacturer's information emphasizes that the drug is novel and acts by a mechanism distinct from the benzodiazepine agonists and antihistamines commonly marketed as hypnotics. The prescribing information does not claim that suvorexant has greater benefits or fewer risks than other drugs marketed for insomnia. Indeed, a search of PubMed ( www.PubMed.gov), ClinicalTrials.gov ( www.ClinicalTrials.gov), and the International Clinical Trials Registry Platform multinational clinical trials registries ( http://www.who.int/ictrp/) found no trials comparing suvorexant with other hypnotics for treatment of insomnia (searched July 17, 2015). Some small comparative trials have been done focused on specific adverse risks such as middle-of-the night impairment and driving impairment ^1^. Physicians and their patients may thus wonder whether they should switch from familiar hypnotics to suvorexant that may have higher costs than popular generics. This discussion presents a clinician's opinions about the choice of hypnotics. Not discussed here are the much more complex issues of when insomnia should be treated with hypnotics and when new developments such as the cognitive-behavioral treatment of insomnia or bright light treatment should be seen as better choices than any hypnotic.

Orexins are excitatory neurotransmitters, secreted primarily by a small number of cells in the lateral hypothalamus ^2– 4^. Orexins have many actions in the brain ^2, 4, 5^, but the current interest is in orexin actions in maintaining wakefulness, for example, through activating tuberomammillary histamine neurons that secrete wake-maintaining histamine throughout many brain areas ^6, 7^. Suvorexant blocks orexin's stimulation of histaminergic neurons. Suvorexant advocates suggests that there is a qualitative difference between suvorexant antagonizing wakefulness whereas in contrast, competitive hypnotics promote sleep, but I cannot conceptualize this distinction clearly. For example, benzodiazepine receptor agonists and histamine receptor antagonists (antihistamines) also suppress histaminergic alerting, besides diverse other actions ^8^. Sleep-wake regulation has been conceptualized as a “flip-flop switch” ^9^ in which a stronger flip or a weaker flop might produce equivalent switching.

When orexin-secreting neurons or orexin receptors are destroyed by autoimmune reactions, narcolepsy may result ^10– 13^. Narcolepsy is an illness characterized by sleep attacks and daytime somnolence, as well as cataplexy (sudden transient weakness or paralysis), sleep paralysis, and hallucinations. The suvorexant inspiration is to help insomnia patients to sleep better by reducing orexigenic maintenance of wakefulness, perhaps similar to what occurs among narcoleptics ^14, 15^, but this idea has limitations. A characteristic of narcolepsy is disturbed nocturnal sleep ^16, 17^. Also, many insomnia patients arise out of bed during the night, and if treated with an orexin receptor antagonist, they might experience certain peculiar narcoleptic symptoms--more about this later. Narcoleptics may not experience more total 24-hour sleep than unaffected people, but more of their sleepiness and sleep tend to occur during the day ^16, 17^. Indeed, narcoleptics suffer daytime somnolence as characterized by a daytime “multiple sleep latency test.” Accordingly, narcolepsy is not usually characterized by a daytime feeling of being well-rested. Because of the relatively long half-life of suvorexant and its day-by-day accumulation, suvorexant might sometimes produce effects like narcolepsy symptoms during the day as well as at night.

Some physicians advise against trying new drugs without proven advantages, until several years of long-term Phase IV monitoring has allowed more experience with the benefits and adverse effects. Let us review some of what is currently known about suvorexant immediate benefits and risks, to offer matters worth considering in making clinical choices in comparison with alternative hypnotics. I shall also emphasize what is unknown, concluding with issues of long-term benefits and risks that may ultimately be far more important than the immediate benefit/risk ratio.

**Table 1.** Abbreviations.

ABBREVIATION	MEANING
ADHD	Attention Deficit Hyperactivity Disorder
AHI	Apnea-Hypopnea Index (respiratory disturbances per hour of sleep)
BMI	Body Mass Index
COPD	Chronic Obstructive Pulmonary Disease
CYP2C19	cytochrome P450 2C19, a liver enzyme acting on drug metabolism
CYP3A	cytochrome P450, family 3, subfamily A: a group of enzymes catalyzing drug metabolism
DEA	United States Drug Enforcement Administration
DORA	dual orexin receptor antagonist
FDA	United States Food and Drug Administration
GABA	Gamma-AminoButyric Acid
Non-REM	sleep other than the REM sleep stage
PSG	PolySomnoGram, generally a sleep recording including EEG
REM	Rapid Eye Movement (sleep stage)
SpO _2_	pulse oximetric arterial saturation of blood, e.g., in percent saturation
T _max_	Time to Maximum drug concentration in blood
URI	Upper Respiratory Infection
WASO	Wake After Sleep Onset, e.g., mid-sleep awakenings or early awakening

## Immediate benefits of suvorexant and alternative hypnotics

Since we do not have comparative controlled trials of suvorexant versus competing hypnotics given for insomnia, the best we can do is to review the evidence of suvorexant benefits versus placebo in randomized double-blind controlled trials. Then we can discuss whether these benefits are likely to be superior or equal to those of popular alternatives, even though randomized unbiased comparative trials are not available.

Many insomnia patients consume hypnotics at bedtime hoping to benefit by better function on the following day. In some studies, suvorexant on average made various kinds of objectively-measured performance such as word recall and driving worse the next morning ^1^, but no significant areas of improved objective function were documented ^1^. If the primary hypnotic benefit desired is to improve next-day performance (measured objectively), suvorexant does not seem to offer that benefit. Quite the opposite. Note that many of the competitive popular hypnotics likewise make an insomnia patient's next-morning performance worse, not better ^18– 20^. It is conceivable that once a hypnotic is fully metabolized (often a variable number of hours after wake-up time), sedation would dissipate and objective performance might rebound. Moreover, considering that insomnia patients sometimes experience increased anxiety after taking a short-acting hypnotic ^21^, and some hypnotics cause increased insomnia on the following night ^22^, afternoon-evening rebound activation and accompanying performance enhancements might conceivably result from some short-acting hypnotics, but this enhancement has not been proven with statistical rigor ^23^ and certainly not with suvorexant. Indeed, I know of no objective evidence that any hypnotic (approved in the U.S.) taken at bedtime improves the next-day performance of insomnia patients. I emphasize objective performance because (like alcoholics), intoxicated hypnotic patients commonly subjectively assert that their performance is enhanced when objective testing shows that it is not.

Prolonged-release melatonin (Circadin®), though not FDA-approved in the U.S., may be an exception to the general failure of sedative-hypnotics to improve next-day performance. Manufacturer-sponsored studies have reported several kinds of performance enhancement ^24, 25^, and there are some reported sleep and behavioral improvements among children with ADHD given ordinary melatonin ^26^.

 Sleep induction strengthens as the suvorexant dosage increases ^1, 27^. Although the manufacturer requested an initial suvorexant dosage ranging from 40 mg down to 15 mg, the FDA would only allow a recommended dose of 10 mg “not to exceed 20 mg daily” ^28^, concluding that a lowered dosage was necessary to reduce the excessive risks produced by higher dosages ^29^. The company's scientists were quoted as telling an FDA committee that “ten milligrams is ineffective,” from a patient’s point of view ^4, 14^. My opinion that 10 mg is generally ineffective agrees with that expressed at that time by the manufacturer. However, desperate to sleep, insomnia patients often take more than the recommended starting dose. Among the first 21 User Reviews of suvorexant listed at the popular WebMD internet site ( www.webmd.com), 2 reported satisfaction with the recommended 10 mg starting dose, 13 reported taking more than 10 mg (as much as 40 mg, sometimes combined with other sedatives), and the others did not report their dosage information ^30^. The FDA authorizes the nocturnal dosage to be increased to 20 mg if 10 mg proves well-tolerated but ineffective. It will be interesting to learn what dosages representative suvorexant patients actually choose to consume.

In the first night of polysomnographic data, 10 mg suvorexant decreased the latency to persistent sleep 3.4 min. (-15.6, 8.7, 95% Confidence Interval) more than placebo, i.e., there was no statistically significant benefit ^1, 27, 31^. Likewise, 20 mg reduced the sleep latency by 9.4 min. (-21.5, 2.9) more than placebo, also not statistically significant, and not clinically significant compared to an initial sleep latency of about 70 minutes. At the end of week 4, 10 mg decreased latency to persistent sleep by 2.3 min. (still not significant), but 20 mg produced 22.3 min. (32.3,12.3) improvement compared to placebo, a statistically significant benefit ^27^. The effects of 10 mg and 20 mg on polysomnographic latency to persistent sleep were found to somewhat greater (and entirely statistically significant) if the preplanned cross-over-phase data of the study were retrospectively excluded ^31^. Also, the 10 mg dose reduced wake after sleep onset (WASO) by about 21 minutes at night 1 and after 4 weeks, which was statistically significant, and the 20 mg dose similarly decreased WASO by 24.7 and 28.1 min. respectively, both significant statistically ^31^. Consequently, the 10 mg dose improved sleep efficiency (percent of in-bed time asleep) by 5.2% on night 1 and 4.7% at the end of week 4, and the 20 mg dose improved sleep efficiency 7.6% and 10.4% respectively, all of which were statistically significant but of uncertain clinical significance, considering that the starting sleep efficiencies were 65%–66% ^27, 31^. By patient self-report, moreover, with 10 mg and 20 mg doses given at night 1 and ending the 4th week, neither the subjective sleep latency nor the subjective total sleep time were improved with statistical or clinical significance ^27^. These patients tended to underestimate the modest objective benefits of suvorexant, so many patients will not be satisfied with either the recommended or the “not to exceed” dosage.

Oddly enough, whereas the patients fairly consistently reported more subjective benefits at the 40 mg dose of suvorexant, that were both statistically and possibly clinically significant benefits (30 mg if age≥65 years), the polysomnographic data for 40 mg showed unimpressive advantages at the end of 4 weeks compared to the lower doses, and the adverse effects were distinctly more common ^1, 27^. This may have been one reason why the FDA insisted on the lower starting dosage.

It is important to keep in mind that the three-month studies described at length in the current Belsomra Prescribing Information ^28, 32^ supported the small-magnitude efficacy of the "not to exceed" dosage of 20 mg (15 mg for age≥65), not the efficacy of the recommended starting dose. The modest efficacies were similar in the three-month studies to those for the 20/15 mg group described in the multiple-dosage study described above. Though statistical significance was more robust in the three-month studies because of the larger group sizes, some of the outcomes still failed to achieve statistical significance at some time points. The recommended 10 mg dose had not been included in the Phase III studies, perhaps another indication that 10 mg was regarded as ineffective. The Phase IIB study described in the two previous paragraphs was the only randomized study reported that compared the 10 mg, 20 mg, and 40 mg doses along with placeboes ^27^.

Overall, comparing suvorexant augmentations of sleep with those reported for the benzodiazepines and benzodiazepine agonists in an authoritative meta-analysis ^33^, all of the hypnotic categories seemed to produce benefits (or lack of benefits) in a similar range. That meta-analysis even questioned whether the “z” hypnotics significantly increased objective total sleep time ^33^. After that meta-analysis, the FDA lowered the recommended doses for zolpidem and eszopiclone, but as with suvorexant, there are few controlled-trial results for the new lower recommended dosages. We do not know if the benefits of low-dose zolpidem and eszopiclone are as minimal as those of suvorexant. For example, the now-recommended 1 mg dosage of eszopiclone was ineffective in many PSG contrasts ^34, 35^. Without randomized comparative trials, one cannot rationally determine whether suvorexant produces as much benefit as the recently-popular hypnotics at currently-recommended doses, since the participants' ages, baseline sleep characteristics, and other factors varied among separate trials, as did elements of the trial designs. One can imagine that suvorexant would be particularly effective for the subgroup of insomnia patients with daytime hyperarousal, but so far no evidence has been produced. I suspect that suvorexant produces better reduction of WASO than popular short-acting hypnotics (although less reduction of sleep latency), but medium-half-life hypnotics such as temazepam and low-dose doxepin might have similar WASO efficacy, and low-dose doxepin may have comparatively fewer adverse effects ^36, 37^. To summarize, for suvorexant, greater overall efficacy than generic competitors at the recommended dosages does not appear likely.

Suvorexant increases nocturnal sleep mainly by reducing WASO, similar to some alternative hypnotics, but unlike short-acting zaleplon, triazolam, or the standard-release zolpidem formulation. The suvorexant effect on the latency to fall asleep is quite weak at the recommended or “not to exceed” dosages due to slow absorption. Accordingly, suvorexant will be particularly unsatisfactory for patients primarily concerned with trouble falling asleep, but suvorexant may be preferred to the shortest-half-life hypnotics for patients who mainly complain of trouble staying asleep and early awakening i.e., WASO. Trouble staying asleep is more common than trouble falling asleep for patients over age 40, probably because circadian rhythms tend to peak progressively earlier from adolescence to old age unless dementia begins.

## Immediate risks of suvorexant and alternative hypnotics

Suvorexant has some distinguishing risks, as well as most of the same immediate risks as the alternative hypnotics. Because suvorexant is not very rapidly absorbed (median T _max_ of 2 hours, range 30 min. to 6 hours, with further delay of approximately 1.5 hours after a high-fat meal) and has an average half-life of approximately 12 hours ^28^, a meaningful blood concentration usually persists throughout the day after prior-evening administration, and there is “an accumulation of approximately 1- to 2-fold with once-daily dosing, leading to an estimated 20% increase in the concentration after repeated dosing ^1, 28^. After 7 nights of administration, the suvorexant blood concentration remained so substantial during the day that just before the next evening dose, the lowest daytime concentration on the 7 ^th^ day was more than half the maximal concentration achieved at T _max_ during the first night ^38^. Since receptor binding and release of orexins is quite indolent, the actions of suvorexant on neurons perhaps lag even later than the plasma T _max_ and the stated half-life might suggest ^4, 39^. Moreover, since suvorexant is mainly metabolized by CYP3A and CYP2C19 enzymes ^28^, the actions of which may be augmented or reduced by common genetic variants ^40^ and other drugs, half-life and daytime accumulation may be quite variable or idiosyncratic. In healthy young adults, the maximum first-night concentrations can vary two-fold, obese females have an approximate 20% increase in morning-after blood levels, and strong CYP3A inhibitors result in three times the drug area under the curve ^1, 38^. Also, suvorexant might influence the metabolism of other drugs through CYP3A. The Prescribing Information recommends against use of suvorexant with strong CYP3A inhibitors ^28^, but one may be skeptical how universally that caution can be observed.

Evidently, the FDA intends that the 5 mg dosage be chosen for those using moderate CYP3A inhibitors or for patients who appear not to tolerate 10 mg well, whereas other patients may need the 20 mg dosage ^29^. Above a 20 mg dosage, the FDA analysis concluded that benefits did not increase in proportion to the strong increase in disturbing adverse effects at the higher dosages. In 30–40 mg dosage groups, 2.8% of patients discontinued use within 3 months due to somnolence, fatigue, sedation, and lethargy combined, and additionally 0.2% also discontinued due to each of the following: nightmares, sleep paralysis, memory impairment, and depression ^1^. In the 15–20 mg groups, the discontinuation rate for adverse events was only 0.6% as compared to 0.4% for placebo ^1^.

As an orexin receptor antagonist, suvorexant appears to produce occasional narcolepsy-like symptoms, especially in the not-recommended 40 mg dosage, such as rare cataplexy (sudden weakness or paralysis), sleep paralysis, hypnagogic or hypnopompic hallucinations, and disturbing dreams ^1^. Suvorexant seems unique among approved hypnotics in its narcolepsy-like adverse effects that can be frightening or temporarily disabling for a very small percentage of patients.

Like most hypnotics with half-lives exceeding 3–6 hours, suvorexant causes daytime somnolence and fatigue among a percentage of users, but suvorexant in recommended doses did not appear to cause reported daytime somnolence more often than alternative hypnotics. In the Phase III trials, some patients suffered disabling sleepiness while driving the following morning. Driving impairments tended to be more severe with zopiclone 7.5 mg than with suvorexant 20 mg or 40 mg (30 mg if age≥65 years), but it was estimated that suvorexant might impair 10%–20% of adult patients on a driving test as much as would a blood alcohol level of 0.05–0.08 ^1^. Note that zopiclone 7.5 mg contains about 3.75 mg eszopiclone, and patients and their physicians approaching such doses must be cautious of potential driving impairment. As with other hypnotics, it may be assumed that this daytime somnolence and these performance impairments can be augmented by combinations of suvorexant with other sedative drugs, narcotics, or alcohol ^38^ that were generally avoided by participants selected for controlled trials. According to the Prescribing Information ^28^, a variety of mental and behavioral impairments may occasionally occur among patients taking suvorexant such as amnesia, anxiety, hallucinations, and complex sleep behaviors. Symptoms of this kind, of which “zombie driving” is an example, occur with other hypnotics and have become somewhat notorious with triazolam and zolpidem ^41– 44^.

In the preapproval trials, suicidal ideation appeared to be a distinct risk of suvorexant, almost entirely at the 30–40 mg dosage level (0.6%) ^1^. That should not be surprising, since suvorexant causes short REM sleep latency ^1^, as is also associated with narcolepsy and depression, and narcolepsy is often treated with antidepressants ^45^. Considering that orexin is increased during pleasure and inhibited during pain, one theory is that a link between narcolepsy and depression results from a changed balance of dynorphin and orexin ^7^. Depression and suicide are likewise associated with many other hypnotics, based on both controlled trials demonstrating causality and epidemiologic studies ^46, 47^.

In a one-year controlled trial of suvorexant 30–40 mg versus placebo, those randomized to suvorexant experienced a dramatic increase in time to sleep onset, once the drug was withdrawn, so that even at the end of two months' drug-free follow-up, the sleep latency of suvorexant-withdrawn patients was subjectively 10–12 min. worse than that of patients who had previously received placebo throughout ^48, 49^. Simply comparing the subjectively-reported sleep of participants while receiving suvorexant vs placebo to the drug-free follow-ups, this withdrawal effect was glaringly apparent. Clinical trial investigators denied that “rebound” was a problem, having relied on a drug “rebound” criterion biased against demonstrating withdrawal effects and lacking statistical power ^50^. Nevertheless, it is to the investigators' credit that they obtained a long two-month post-drug follow-up. This was the longest-lasting randomized, controlled demonstration of hypnotic-withdrawal insomnia of which I am aware ^48^. Certainly, popular alternative hypnotics also produce drug-withdrawal insomnia ^22, 49^, but their withdrawal liabilities have not been studied with equivalent designs. Zolpidem 10 mg caused no appreciable problem in a 1-year study of somewhat different design with a somewhat anomalous outcome ^51^. We do not know which drugs would cause more withdrawal distress given at the recommended dosages.

Some hypnotics cause increased infections in randomized controlled trials, supported by extensive epidemiology ^52– 55^. When given suvorexant, patient infections and infestations overall were about equal with placebo, but there was a dose-response trend for more common "URI" reports among participants receiving suvorexant than placebo ^1^. The controlled trial evidence for causing infections would appear stronger for alternative hypnotics than for suvorexant.

To examine effects of suvorexant on nocturnal respiration, patients with COPD and "moderate" sleep apnea were randomized to suvorexant 40 mg (30 mg if age≥65 years) or placebo for 4-night sleep recordings. Participants had a mean SpO _2_ of >94% awake and >93% during sleep, and mean BMI of 25.9. Nevertheless, suvorexant produced significantly reduced SpO _2_ both during wake and during sleep, and increased time below 85% SpO _2_, though these effects were quite small and were not considered clinically significant ^56^. In a study of participants with "mild or moderate" sleep apnea and with average age 49, night 4 AHI (apnea-hypopnea index) was increased from 14.41 to 17.07 events per hour with suvorexant versus placebo, a difference of 2.66 (0.22 to 5.09), therefore significant ^57^. Though these adverse effects did not appear clinically significant on average, in both studies suvorexant did impair nocturnal breathing. These were not the sorts of patients whose nocturnal breathing would be most vulnerable to a hypnotic and of greatest concern, e.g., those with marked nocturnal oxygen desaturation, obesity, and concomitant use of narcotics ^58^ or other sedatives. Since alternative hypnotics also depress nocturnal respiration, it is unclear if suvorexant causes more respiratory risk than the alternatives.

Falls are strongly associated with use of many hypnotics ^59– 61^, but falls among patients randomized to suvorexant were no more common than those among participants randomized to placebo ^1^.

Like most benzodiazepine-agonist hypnotics, suvorexant is thought to have some addiction potential and is rated Schedule IV by the DEA ^62^. In contrast, doxepin, antihistamines, and melatonin are not controlled by the DEA.

To summarize, it seems unlikely that suvorexant could prove superior to alternative hypnotics in comparative trials focusing on the immediate benefits/risks ratios, because of 1) weak subjective benefit at low doses, 2) weak polysomnographic benefit for reducing sleep latency, 3) a relatively long half-life resulting in accumulation and daytime sedation, 4) particularly variable rates of absorption and CYP3A metabolism making dosing unpredictable, and 5) relatively unique narcolepsy-like symptoms with more-than-recommended doses. On the other hand, the long-term effects of hypnotics might be more important than their immediate effects.

## Long-term benefits and risks of suvorexant and alternative hypnotics

Most patients who receive a prescription for a hypnotic consume the drug for only a brief time. However, the unusual patient who consumes a hypnotic nightly or several times a week for years receives so many prescriptions, that these heavy users consume most of the hypnotic drug market ^63, 64^. Among long-term habitual hypnotic consumers, there is a need to consider hypnotic benefits and risks not only for sleep but also for the long-term risks of dementia, cancer, and mortality. Unfortunately, there have been no long-term controlled trials assessing years of hypnotic usage by contrasting samples randomized to a hypnotic versus placebo, or comparing different hypnotics randomly assigned. Trials of cardiology drugs such as statins or the Women's Health Initiative long-term trial of estrogens assessed years of drug usage among tens of thousands of participants, but we have no comparable controlled trials of hypnotics.

The body tends to clear amyloid-β from brain intercellular regions during sleep, a process that may be inhibited during wakefulness by orexin ^65– 69^. This has led to speculation that orexin antagonists, such as suvorexant, might hypothetically reduce risks of Alzheimer's disease. However, one small study found evidence of an average amount of Alzheimer's amyloid plaque accumulation in brains of aged narcoleptics ^70^. Further, suvorexant in the recommended dosages increases total sleep rather little, and the increment is mainly REM sleep rather than deep sleep ^4^. Since it appears to be non-REM sleep that is associated with amyloid-β clearance rather than orexin itself ^68^, any idea that suvorexant would have a beneficial effect on amyloid-β may be wishful thinking ^70^. In contrast, there is more persuasive evidence that prior use of benzodiazepine-agonist hypnotics is associated with future Alzheimer's dementia ^71– 73^. Causality has not been proven.

There is suggestive evidence from small controlled trials that benzodiazepine-agonist hypnotics cause cancer. In a group of rather small controlled trials reviewed by the FDA, 13 incident cancer cases (mainly skin cancers) were found among patients randomized to hypnotics, but none were found among the sometimes-smaller randomized placebo groups ^74^. Further, epidemiologic studies have supported an association of prior hypnotic use with cancer incidence ^64, 75, 76^. It is controversial whether the presence of epidemiologic association might imply that benzodiazepine-agonist hypnotics cause cancer ^77, 78^. In the distinct case of suvorexant, my tabulation of the suvorexant randomized controlled trials reported to ClinicalTrials.gov ( www.ClinicalTrials.gov) indicated no incident malignancies among 493 participants receiving suvorexant 20 mg (15 mg if age≥65 years), 9 incident malignancies among 1291 participants receiving suvorexant 40 mg (30 mg if age≥65 years), and 9 malignancies among 1025 participants randomized to placebo. Thus, incident cancers were less frequent among those randomized to suvorexant, particularly less than 30 mg as compared to placebo. These differences in cancer incidence were not statistically reliable in the suvorexant trials for these very infrequent cancer events. The FDA's approach to enumerating the incident neoplasms in suvorexant controlled trials produced slightly different tabulations, but essentially similar trends were described ^1^. In summary, more data are needed, but there is a possibility that benzodiazepine agonist hypnotics are carcinogenic whereas suvorexant is not. Even the possibility that suvorexant is anti-neoplastic cannot be excluded.

Finally, there are now more than 20 epidemiologic studies showing that use of benzodiazepine-agonists and diphenhydramine has been significantly associated with excess mortality, with hazard ratios as high as 3 to 5 ^64, 79– 81^. A much smaller number of studies has observed no significant survival risk associated with hypnotics use, but no published studies yet have suggested any evidence that use of hypnotics improves survival. Indeed, some studies suggest that hypnotics have posed as much mortality risk as cigarettes ^64, 79, 82^. Despite various efforts of many investigators to control for potential confounding in epidemiologic studies, it remains possible that this strong risk association is entirely due to statistical confounding, reflecting no causality ^83^. A persuasive demonstration of mortality causation could only come from long-term randomized controlled trials or perhaps Mendelian randomization studies. At present, there is no epidemiologic evidence whether suvorexant use is associated with increased or decreased survival. There is as yet no evidence base permitting a guess about how suvorexant compares with alternative hypnotics for association with mortality.

## Conclusions

With the limited available evidence, we can only guess about whether suvorexant is more or less effective and more or less safe than popular prescription hypnotics. However, none of them are very effective for increasing objective sleep or for improving daytime performance. Apart from some very small unpublished Phase II trials focused on special risks, there have been no randomized comparative trials examining whether the suvorexant benefits/risks ratio is better or worse than that of popular alternatives, so there are no bases for a clear preference save the amount of prior experience and costs. One might suppose that had the developers thought that suvorexant was superior in its immediate effects, they would have performed comparative clinical trials to highlight the advantages. Currently, suvorexant appears more expensive than many popular generic hypnotics. Suvorexant has so-far undergone little Phase IV safety surveillance. It appears that the overall balance of immediate benefits and risks with suvorexant most likely would be comparable or inferior to alternative hypnotics such as zolpidem ( Table 2). As to long-term benefits and risks, we will have to hope that the industry conducts the necessary long-term comparative trials to assess which hypnotic compounds are safest and most beneficial.

Table 2 summarizes some evidence concerning how suvorexant might compare with zolpidem, currently the most popular hypnotic in the United States. The comparisons for long-term risks and benefits are pure speculation.

**Table 2.** Comparison of suvorexant and zolpidem: likely benefits and risks.

	possibly suvorexant better	possibly zolpidem better	not enough data to guess
**Immediate Benefits**			
decreased sleep latency		X	
decreased wake after sleep onset	X		
increased total nightly sleep			X
**Immediate Risks**			
daytime sleepiness and fatigue			X
impaired driving		X	
impaired performance		X	
narcoleptiform symptoms		X	
amnesia, anxiety, hallucinations			X
complex sleep behaviors			X
depression and suicidal thoughts			X
withdrawal insomnia		X	
infections	X		
respiratory depression			X
falls	X		
addiction/abuse potential			X
**Long-term Risks & Benefits**			
dementia	?		
cancer	?		
mortality vs survival			?

**X** Based on somewhat-parallel placebo-controlled-trials studies but no comparative-trials studies

**?** Based only on non-comparative epidemiology and scientific speculation

## The future choice of the best hypnotics

There are other orexin receptor antagonists in development. Possibly, a new orexin receptor antagonist will become available as a hypnotic with more reliable pharmacokinetics than suvorexant and a shorter T _max_ and half-life. Perhaps such a drug might safely be given in a more effective bedtime dosage, with less danger of daytime adverse effects. A focus of future study will be how orexin receptor antagonists compare with alternative treatments of disturbed sleep such as cognitive behavioral treatment of insomnia or bright light treatment. There is a growing consensus that current data favor cognitive-behavioral therapy over hypnotics, and bright light may be superior for particular circadian rhythm sleep disorders manifesting as insomnia.

Looking forward, I anticipate that better-organized exploitation of electronic medical records will produce increasing scrutiny of effects of hypnotics on inpatient falls, infections, and length of stay. Likewise, there will be increasing examination of the association of hypnotics with outpatient readmissions, infections, dementia, cancer, and mortality. Increasing use of genome-wide-association studies, exome sequencing, and whole-genome sequencing will make it possible to do Mendelian randomization studies that assess the causality of hypnotic associations with excess depression, infection, cancer, and mortality. Unfortunately, it will be many years before experience will be collected sufficient to apply Mendelian randomization strategies to orexin receptor antagonists. It is only speculation that in regard to long-term risks, particularly dementia, cancer, and mortality, suvorexant might be found safer than alternatives or even beneficial. Unless and until the industry can provide us with long-term-trials evidence of more distinct suvorexant advantages, cautious and cost-conscious physicians and their patients may prefer the alternatives.
