# Supplementary material for: Is suvorexant a better choice than alternative hypnotics?
Source: F1000Res. 2015 Aug 3;4:456. [Version 1] doi: 10.12688/f1000research.6845.1 (PMC4648222; doi:10.12688/f1000research.6845.1)
Supplement: Peer review history

# Peer review: Referee response for version 1

**DOI:** 10.5256/f1000research.7359.r10375

**Published:** 2015-09-18

**Authors:** Børge Sivertsen

**Affiliations:** 1 Division of Mental Health, Norwegian Institute of Public Health, Bergen, Norway

- **Title and Abstract**: The title and abstract is well formulated and indeed representative for the rest of the full text paper. Being an opinion article, it is made clear from the beginning – including the abstract - that no new original data are presented; rather an expert’s summarization of available evidence regarding the use of suvorexant.
- **Article content**: Dr. Kripke provides a very thorough review of available literature, both published and unpublished reports, with regards to various aspects of suvorexant. Existing evidence (or lack thereof) of both efficacy, side-effects, risk/benefit ratio, comparisons studies etc., are clearly presented, and the conclusions drawn from these reports are well-balanced.
- **Conclusions**: The conclusions are very clearly stated, yet sensible, balanced as well as justified on the basis of the available data regarding suvorexant. I consider this an important contribution that will improve our understanding, and provide a solid scientific foundation useful for both clinicians and researchers alike.

I have read this submission. I believe that I have an appropriate level of expertise to confirm that it is of an acceptable scientific standard.

# Peer review: Referee response for version 1

**DOI:** 10.5256/f1000research.7359.r9764

**Published:** 2015-08-05

**Authors:** Jerome Siegel

**Affiliations:** 1 Department of Psychiatry and Biobehavioral Sciences, School of Medicine, University of California, Los Angeles, Los Angeles, CA, USA

Kripke presents a thorough analysis of the risks and possible benefits of suvorexant for the treatment of insomnia. Suvorexant is an antagonist for the two receptors for hypocretin (also called orexin), a peptide released by a small group of neurons in the hypothalamus. Soon after the peptide was identified, it was found that 90% of neurons containing this peptide are lost in human narcolepsy ^1^^,^^2^. Since one of the main symptoms of narcolepsy is sleepiness, it seemed plausible that a drug blocking hypocretin receptors would cause sleepiness, an effect that might be useful in treating insomnia. Suvorexant is the first such drug to hit the market. Human insomnia is a complaint about inadequate sleep, but is not necessarily correlated with low sleep duration or with decreased lifespan ^3^^,^^4^.

Current insomnia treatments act on GABA receptors, particularly on the benzodiazepine type of GABA receptor. An obvious problem with manipulation of systemic GABA levels is the very large number of GABA neurons and GABA receptors in the brain. GABA receptors exist not only in regions such as the anterior hypothalamus and adjacent forebrain regions implicated in sleep induction, but throughout the brain.  In some regions nearly 90% of neurons contain GABA ^5^. Benzodiazepine receptors also exist in large numbers in bodily organs including the heart ^6^^,^^7^, gall bladder, urinary bladder ^8^, thyroid, liver ^9^, lung, stomach ^10^^,^^11^, testes ^11^, pancreas ^10^ and kidneys ^10^^,^^12^ and are activated by many commonly used sleeping pills ^13^^,^^14^. Benzodiazepine receptors are present on red blood cells, on tumors, as well as on cells of the immune system ^15^^-^^19^. Increased rates of infection have been reported with the use of hypnotics ^20^.

In contrast to GABA, there only about 75,000 hypocretin neurons in the human brain ^2^^,^^21^^,^^22^, a tiny fraction of the 75,000,000,000 neurons estimated to be in the human brain. They are distributed from the most medial portions of the hypothalamus adjacent to the 3 ^rd^ ventricle, to the far lateral hypothalamus. Although initial reports suggested that there were orexin neurons in the gut, these reports have not been replicated ^23^. Hypocretin neurons have widespread projections, directly innervating and activating cortical, subcortical and brainstem neurons ^24^.  

Some work has suggested potential problems with dual orexin receptor antagonists. Humans who have attempted suicide have reduced levels of hypocretin-1 in their cerebrospinal fluid ^25^. In a study of human patients with electrodes implanted in the amygdala for diagnostic purposes, we found that hypocretin release was maximal during pleasure and was minimal when they reported feeling sad or when they were in pain, despite a high level of arousal ^26^. Allowing for species differences, these human data bear considerable resemblance to data on hypocretin neuron activity in animals. In normal mice, we found that hypocretin neurons are maximally active during performance of rewarded behaviors ^27^ and that hypocretin knockout mice were strikingly deficient in staying awake to perform rewarded behaviors. Our studies of Fos expression in wild type mice also showed that hypocretin neurons were not activated beyond baseline levels during foot shock, or foot shock avoidance behavior, despite high levels of EEG arousal ^27^. We have also reported that hypocretin neuronal activity in rats is suppressed in novel situations eliciting withdrawal, despite maximal levels of EEG activation. In contrast, hypocretin neuron  activity is high during grooming and exploration ^28^. The animal and human studies indicate that hypocretin cells are not simply related to arousal, but are strongly related to positive emotions. This is consistent with the evidence that human depression and reported difficulties with social interaction in narcolepsy may result from the loss of hypocretin function, as would occur with receptor antagonists ^29^^-^^34^. 

These considerations suggest that depression and even suicide might be a risk from the use of orexin receptor antagonists. However, this need not be the case if the drug induces sleep rapidly, and does not persist in the brain. Kripke reviews evidence suggesting that chronic use produces longterm inactivation of hypocretin receptors, highlighting this risk.

I have read this submission. I believe that I have an appropriate level of expertise to confirm that it is of an acceptable scientific standard.

## References

[1] PeyronCFaracoJRogersWRipleyBOvereemSCharnayYNevsimalovaSAldrichMReynoldsDAlbinRLiRHungsMPedrazzoliMPadigaruMKucherlapatiMFanJMakiRLammersGJBourasCKucherlapatiRNishinoSMignotE: A mutation in a case of early onset narcolepsy and a generalized absence of hypocretin peptides in human narcoleptic brains. *Nat Med.*.2000;6(9) : 10.1038/79690 991-997 10.1038/79690 10973318

[2] ThannickalTCMooreRYNienhuisRRamanathanLGulyaniSAldrichMCornfordMSiegelJM: Reduced number of hypocretin neurons in human narcolepsy. *Neuron*.2000;27(3) : 10.1016/S0896-6273(00)00058-1 469-474 10.1016/S0896-6273(00)00058-1 11055430PMC8760623

[3] BuysseDJ: Insomnia. *JAMA*.2013;309(7) : 10.1001/jama.2013.193 706-716 10.1001/jama.2013.193 Reference source 23423416PMC3632369

[4] KripkeDFGarfinkelLWingardDLKlauberMRMarlerMR: Mortality associated with sleep duration and insomnia. *Arch Gen Psychiatry*.2002;59(2) : 10.1001/archpsyc.59.2.131 131-136 10.1001/archpsyc.59.2.131 Reference source 11825133

[5] ZhaoCEisingerBGammieSC: Characterization of GABAergic neurons in the mouse lateral septum: a double fluorescence in situ hybridization and immunohistochemical study using tyramide signal amplification. *PLoS One*.2013;13(8) : 10.1371/journal.pone.0073750 e73750 10.1371/journal.pone.0073750 Reference source 23967349PMC3742568

[6] LiJXiaoJLiuYZhangGZhangHLiangDLiuYZhangYHuYYuZYanBJiangBPengLZhouZNChenYH: Mitochondrial benzodiazepine receptors mediate cardioprotection of estrogen against ischemic ventricular fibrillation. *Pharmacol Res.*.2009;60(1) : 10.1016/j.phrs.2009.03.003 61-67 10.1016/j.phrs.2009.03.003 Reference source 19427588

[7] BrownDAAonMAAkarFGLiuTSorarrainNO'RourkeB: Effects of 4'-chlorodiazepam on cellular excitation-contraction coupling and ischaemia-reperfusion injury in rabbit heart. *Cardiovasc Res.*.2008;79(1) : 10.1093/cvr/cvn053 141-149 10.1093/cvr/cvn053 Reference source 18304929PMC2562874

[8] KumarAMuzikOChuganiDChakrabortyPChuganiHT: PET-derived biodistribution and dosimetry of the benzodiazepine receptor-binding radioligand (11)C-(R)-PK11195 in children and adults. *J Nucl Med*.2010;51(1) : 10.2967/jnumed.109.066472 139-144 10.2967/jnumed.109.066472 Reference source 20008990

[9] LuotoPLaitinenISuilamoSNågrenKRoivainenA: Human dosimetry of carbon-11 labeled N-butan-2-yl-1-(2-chlorophenyl)-N-methylisoquinoline-3-carboxamide extrapolated from whole-body distribution kinetics and radiometabolism in rats. *Mol Imaging Biol.*.2010;12(4) : 10.1007/s11307-009-0293-1 435-442 10.1007/s11307-009-0293-1 Reference source 19941083

[10] TyagiNLominadzeDGillespieWMoshalKSSenURosenbergerDSSteedMTyagiSC: Differential expression of gamma-aminobutyric acid receptor A (GABA(A)) and effects of homocysteine. *Clin Chem Lab Med*.2007;45(12) : 10.1515/CCLM.2007.342 1777-1784 10.1515/CCLM.2007.342 Reference source 17990949PMC3182488

[11] VersijptJDumontFThierensHJansenHDe VosFSlegersGSantensPDierckxRAKorfJ: Biodistribution and dosimetry of [123I]iodo-PK 11195: a potential agent for SPET imaging of the peripheral benzodiazepine receptor. *Eur J Nucl Med.*.2000;27(9) : 10.2967/jnumed.109.066472 1326-1333 10.2967/jnumed.109.066472 11007514

[12] HauetTHanZWangYHameuryFJayleCGibelinHGoujonJMEugeneMPapadopoulosV: Modulation of peripheral-type benzodiazepine receptor levels in a reperfusion injury pig kidney-graft model. *Transplantation*.2002;74(11) :1507-1515 Reference source 1249078210.1097/00007890-200212150-00006

[13] VeenmanLGavishM: The peripheral-type benzodiazepine receptor and the cardiovascular system. Implications for drug development. *Pharmacol Ther*.2006;110(3) : 10.1016/j.pharmthera.2005.09.007 503-524 10.1016/j.pharmthera.2005.09.007 Reference source 16337685

[14] SannaEBusoneroFTalaniGCartaMMassaFPeisMMacioccoEBiggioG: Comparison of the effects of zaleplon, zolpidem, and triazolam at various GABA(A) receptor subtypes. *Eur J Pharmacol.*.2002;451(2) : 10.1016/S0014-2999(02)02191-X 103-110 10.1016/S0014-2999(02)02191-X Reference source 12231378

[15] MiltykWPalłkaMKarnaEJarzabekKBoujradNKnappP: Antimitotic activity of high affinity ligands for peripheral benzodiazepine receptor (PBR) in some normal and neoplastic cell lines. *Adv Med Sci.*.2006;51:156-159 17357298

[16] AlamSLaughtonDLWaldingAWolstenholmeAJ: Human peripheral blood mononuclear cells express GABAA receptor subunits. *Mol Immunol.*.2006;43(9) : 10.1016/j.molimm.2005.07.025 1432-1442 10.1016/j.molimm.2005.07.025 Reference source 16213022

[17] CostaBSalvettiARossiLSpinettiFLenaAChelliBRechichiMDa PozzoEGremigniVMartiniC: Peripheral benzodiazepine receptor: characterization in human T-lymphoma Jurkat cells. *Mol Pharmacol*.2006;69(1) : 10.1124/mol.105.015289 37-44 10.1124/mol.105.015289 Reference source 16189298

[18] SiegelJM: The neurobiology of sleep. *Semin Neurol.*.2009;29(4) : 10.1055/s-0029-1237118 277-296 10.1055/s-0029-1237118 Reference source 19742406PMC8809119

[19] LeeDHKangSKLeeRHRyuJMParkHYChoiHSBaeYCSuhKTKimYKJungJS: Effects of peripheral benzodiazepine receptor ligands on proliferation and differentiation of human mesenchymal stem cells. *J Cell Physiol*.2004;198(1) : 10.1002/jcp.10391 91-99 10.1002/jcp.10391 Reference source 14584048

[20] JoyaFLKripkeDFLovingRTDawsonAKlineLE: Meta-analyses of hypnotics and infections: eszopiclone, ramelteon, zaleplon, and zolpidem. *J Clin Sleep Med*.2009;5(4) :377-383 Reference source 19968019PMC2725260

[21] ThannickalTCNienhuisRSiegelJM: Localized loss of hypocretin (orexin) cells in narcolepsy without cataplexy. *Sleep*.2009;32(8) :993-998 Reference source 1972525010.1093/sleep/32.8.993PMC2717206

[22] ThannickalTCLaiYYSiegelJM: Hypocretin (orexin) cell loss in Parkinson's disease. *Brain*.2007;130(pt 6) : 10.1093/brain/awm097 1586-1595 10.1093/brain/awm097 Reference source 17491094PMC8762453

[23] BaumannCRClarkELPedersenNPHechtJLScammellTE: Do enteric neurons make hypocretin?. *Regul Pept.*.2008;147: 10.1016/j.regpep.2007.11.006 1-3 10.1016/j.regpep.2007.11.006 Reference source 18191238PMC2276606

[24] PeyronCTigheDKvan den PolANde LeceaLHellerHCSutcliffeJGKilduffTS: Neurons containing hypocretin (orexin) project to multiple neuronal systems. *J Neurosci*.1998;18(23) :9996-10015 Reference source 982275510.1523/JNEUROSCI.18-23-09996.1998PMC6793310

[25] BrundinLBjörkqvistMTräskman-BendzLPetersénA: Increased orexin levels in the cerebrospinal fluid the first year after a suicide attempt. *J Affect Disord.*.2009;113(1-2) : 10.1016/j.jad.2008.04.011 179-182 10.1016/j.jad.2008.04.011 Reference source 18533274

[26] BlouinAMFriedIWilsonCLStabaRJBehnkeEJLamHAMaidmentNTKarlssonKÆLapierreJLSiegelJM: Human hypocretin and melanin-concentrating hormone levels are linked to emotion and social interaction. *Nat Commun.*.2013;4: 10.1038/ncomms2461 1547 10.1038/ncomms2461 Reference source 23462990PMC3595130

[27] McGregorRWuMFBarberGRamanathanLSiegelJM: Highly specific role of hypocretin (orexin) neurons: differential activation as a function of diurnal phase, operant reinforcement versus operant avoidance and light level. *J Neurosci.*.2011;31(43) : 10.1523/JNEUROSCI.4017-11.2011 15455-15467 10.1523/JNEUROSCI.4017-11.2011 Reference source 22031892PMC3230273

[28] MileykovskiyBYKiyashchenkoLISiegelJM: Behavioral correlates of activity in identified hypocretin/orexin neurons. *Neuron*.2005;46(5) : 10.1016/j.neuron.2005.04.035 787-798 10.1016/j.neuron.2005.04.035 Reference source 15924864PMC8281334

[29] ZamarianLHöglBDelazerMHingerlKGabeliaDMitterlingTBrandauerEFrauscherB: Subjective deficits of attention, cognition and depression in patients with narcolepsy. *Sleep Med*.2015;16(1) : 10.1016/j.sleep.2014.07.025 45-51 10.1016/j.sleep.2014.07.025 Reference source 25434299

[30] InocenteCOGustinMPLavaultSGuignard-PerretARaouxAChristolNGerardDDauvilliersYReimãoRBat-PitaultFLinJSArnulfILecendreuxMFrancoP: Depressive feelings in children with narcolepsy. *Sleep Med*.2014;15(3) : 10.1016/j.sleep.2013.08.798 309-314 10.1016/j.sleep.2013.08.798 24503472

[31] OhayonMM: Narcolepsy is complicated by high medical and psychiatric comorbidities: a comparison with the general population. *Sleep Med*.2013;14(6) : 10.1016/j.sleep.2013.03.002 488-492 10.1016/j.sleep.2013.03.002 23643648

[32] DimitrovaAFronczekRVan der PloegJScammellTGautamSPascual-LeoneALammersGJ: Reward-seeking behavior in human narcolepsy. *J Clin Sleep Med.*.2011;7(3) : 10.5664/JCSM.1076 293-300 10.5664/JCSM.1076 21677900PMC3113969

[33] MamelakM: Narcolepsy and depression and the neurobiology of gammahydroxybutyrate. *Prog Neurobiol.*.2009;89(2) : 10.1016/j.pneurobio.2009.07.004 193-219 10.1016/j.pneurobio.2009.07.004 Reference source 19654034

[34] Szklo-CoxeMYoungTFinnLMignotE: Depression: relationships to sleep paralysis and other sleep disturbances in a community sample. *J Sleep Res.*.2007;16(3) : 10.1111/j.1365-2869.2007.00600.x 297-312 10.1111/j.1365-2869.2007.00600.x Reference source 17716279PMC2800990
